# Simultaneous Screening of Six Families of Antibiotic Residues in Milk Samples by Biochip Multi-array Technology

**DOI:** 10.5812/ijpr-136363

**Published:** 2023-08-20

**Authors:** Hassan Yazdanpanah, Mahraz Osouli, Jamshid Salamzadeh, Elham Rashidi, Zakieh Karimi, Leila Beykmohammadi, Samira Eslamizad

**Affiliations:** 1 Food Safety Research Center, Shahid Beheshti University of Medical Sciences, Tehran, Iran; 2 Department of Toxicology and Pharmacology, School of Pharmacy, Shahid Beheshti University of Medical Sciences, Tehran, Iran; 3 School of Pharmacy, Shahid Beheshti University of Medical Sciences, Tehran, Iran

**Keywords:** Milk, Screening, Validation, Quinolones, Tylosin, Ceftiofur, Streptomycin, Tetracycline, Norfloxacin, Florfenicol, Multi-array, Biochip

## Abstract

**Background:**

Antimicrobial compounds are used in animal husbandry to prevent and treat bacterial diseases and as illegal growth-promoting agents. Due to the excessive and inappropriate use of antibiotics, the antibiotic residues in milk can cause allergic reactions and antibiotic resistance. A rapid biochip-based method for the multi-analyte screening of 6 families of antibiotic residues (quinolones, ceftiofur, florfenicol, streptomycin, tylosin, and tetracyclines) in milk was validated based on Commission Decision 2002/657 and the European guidance for screening methods for veterinary medicinal products.

**Methods:**

This methodology allows the 6 antibiotic families to be detected simultaneously, increasing the screening capacity and reducing costs in test settings. The method's applicability was shown by screening 38 UHT cow milk samples taken from Tehran province, IR Iran.

**Results:**

The results showed that the positive threshold T was above Fm, and the CCβ was below the European Commission's Maximum Residue Limit (MRL) (100 ppb for ceftiofur and tetracycline and 50 ppb for tylosin in milk). Norfloxacin was detected in about 8% of the samples and tylosin in 2.63%. The total antibiotic concentration in UHT cow milk samples was lower than the European Commission's MRL.

**Conclusions:**

This study showed that the biochip technique is valid for screening tylosin, ceftiofur, streptomycin, tetracycline, norfloxacin, and florfenicol in milk. It was found that the method was easy, quick, and capable of detecting 6 families of antibiotic residues simultaneously from a single milk sample without sample preparation.

## 1. Background

Milk is one of the most important forms of animal-source foods. It is a good and complete source of energy, protein, and micronutrients promoting growth (1). Adequate consumption of milk and its derivatives is presumably beneficial for all ages. Prevention of overweight, obesity, diabetes, and cardiovascular disease are some of its benefits in literature (2). With the development of veterinary pharmacies, the emergency, and improvement of veterinary drugs will likely positively affect the livestock and poultry industry, such as disease diagnosis, control, and prevention. However, some farmers may use high-dose veterinary drugs as growth promoters to generate higher profits, posing a potential health risk to customers (3-5).

The quality and safety of animal-source food is becoming a worldwide public health problem since using antibiotics in animals has found a key role in industrial livestock (6). The antibiotics used in livestock are ingested by humans when they consume foods (7). Long-term ingestion of animal products contaminated with antibiotic residues would have irreversible effects on human health. More specifically, the effects may involve increasing antibiotic resistance in bacteria, allergic reactions, toxicity, anaphylaxis, carcinogenesis, mutagenicity, teratogenicity, malformations, and imbalance of the intestinal bacterial colony. In addition, leakage or leakage of unprocessed antibiotics into the natural environment can negatively affect the ecosystem. Finally, food safety problems resulting from improper handling and detection of veterinary drug residues can hamper the development of the livestock industry and the world economy (3, 8).

For consumer safety, the Food and Drug Administration, European Union (EU), and other international regulators have established maximum residue limits (MRLs) for certain antibiotics in foods of animal origin, including milk (9-11). Given these MRLs, sensitive and selective analysis methods are strongly recommended for detecting low concentrations of these compounds in milk samples (10).

The fast and efficient detection of drug residues in animal foods remains a key issue. Several methods for detecting residues have been described, such as microbiological methods, Enzyme-linked Immunosorbent Assay (ELISA), and chromatographic methods (12, 13). High-sensitivity instrumental methods are commonly employed as confirmatory techniques. However, these detection methods necessitate sophisticated instruments and highly skilled personnel. Besides, complicated sample pretreatment steps are always needed, severely limiting their wide application (14). Microbiological methods in high-throughput residue screening perform well but lack specificity and sensitivity. Conventional immunoassays are inexpensive and highly sensitive but often require experienced personnel (14). The ELISA method is simple, sensitive, and inexpensive; however, only one class of drugs can be detected at a time, and the detection efficiency is not high (12). Biosensor technology based on antibody chips perfectly meets this requirement. By enriching different antigen-antibody reactions on the chip and capturing immunofluorescence sensor signal values of different chips, it is realized that multiple drugs can be examined simultaneously in many samples (12). Biochip array technology is a kind of immunoassay-based technology enabling the semi-quantitative simultaneous determination of multiple analytes in samples using miniaturized immunoassays applied on the semi-automated analyzer called Evidence Investigator (15). Microarray technology is a powerful analytical tool for simultaneously detecting multiple analytes in a single sample and is an emerging field in analytical chemistry. A microarray consists of a reactive dot matrix on a supporting material (13).

This study validates a biochip array technology for the simultaneous detection of 6 families of antibiotic residues (quinolones, ceftiofur, florfenicol, streptomycin, tylosin, and tetracyclines) in milk and utilizes this procedure on real milk samples. This methodology was validated following European Decision No. CE/2002/657 (16) and the European guidance document for the validation of methods for the detection of residues of veterinary medicinal products (17). The procedure was applied to 38 UHT cow milk samples collected from the Iranian market.

## 2. Methods

### 2.1. Chemicals and Reagents

Norfloxacin, ceftiofur (CEFT), florfenicol (FFL), streptomycin sulfate salt (STR), tylosin (TYL), and tetracycline (TCN) were purchased from Sigma-Aldrich (Germany). Antimicrobial Array II kit (EV 3524A) and milk preparation kit (EV 3776) were obtained from Randox Food Diagnostics (UK).

### 2.2. Apparatus

We employed an Evidence Investigator biochip analyzer (Randox Food Diagnostics, UK), vortex model Hei-MIX Reax top (Heidolph, Germany), centrifuge Rotinta 380R (Hettich, Germany), and roller mixer model BMW (Behdad, IRAN).

### 2.3. Blank and Real Samples

Different batches of cow milk were obtained, each with varying fat levels and shelf life. These included long-life and skimmed milk, as well as fresh and long-life Bio-milk containing 3.5% fat. Samples of milk were collected from the UK and Austria and analyzed to ensure they did not contain any residues of the 6 families of antibiotics (quinolones, ceftiofur, florfenicol, streptomycin, tylosin, and tetracyclines).

Thirty-eight UHT cow milk samples [15 low-fat milk samples (1.5%), 17 half-fat milk samples (2.5%), and 6 whole milk samples (3%)] were purchased at retail stores between July and August 2017. The milk samples were stored at 2 - 8ºC until analysis.

### 2.4. Preparing Standard Solutions

Stock solutions of all antibiotics were prepared at a concentration of 1 mg/mL in methanol, except for norfloxacin which was soluble in 1 M NaOH, ceftiofur in a mixture of methanol and DMSO (1:1), and streptomycin in water. The stock solutions were diluted with their solvents to make the intermediate standards (10 ng/mL). Then, the intermediate standard solutions were diluted to obtain working solutions for each compound.

### 2.5. Sample Preparation

Before analysis, no special preparation exists for milk samples, except for semi-skimmed and full-fat milk to eliminate the fat by centrifuging the milk (10 min at 2,880 rcf). Skim milk samples do not need to be centrifuged prior to the run. Spiked samples were prepared from the working solution by diluting with blank milk at different spiking levels (1:9).

### 2.6. Multi-array Technology

The Evidence Investigator system is based on the biochip, which contains a set of Discrete Test Regions (DTRs) of immobilized antibodies specific to various antibiotics. A competitive chemiluminescent immunoassay format was used. In relative light unit (RLU), higher antimicrobial concentrations in a sample would reduce the binding of labeled antimicrobials with horseradish peroxidase (PRH), decreasing the chemiluminescence signal emitted (18).

Antimicrobial Array II was applied to the Evidence Investigator biochip analyzer. Nine biochips exist in a biochip carrier. The biochips are also the vessels where simultaneous immunoreactions occur. The experimental procedure followed the manufacturer's guidelines. Briefly, 100 µL of milk buffer was added to each calibrator and control biochip, and 100 µL of assay buffer was added to the biochips assigned for sample analysis. Then, 100 µL of calibrator or sample was added to each biochip. All sides of the tray, which can hold up to 6 biochip carriers, have been gently tapped to mix the reagents. Next, the handling tray was incubated for 30 minutes at 25°C and 370 rpm in the thermoshaker provided. Next, 100 µL of working strength conjugate was added to each biochip, and the tray was incubated for another 60 minutes at 25°C and 370 rpm. Following incubation, the liquid was discarded, wells were washed with diluted wash buffer, and the residual liquid was removed by lint-free tissue. Next, 250 µL of working signal reagent was added to each well and coated to protect against light. After 2 minutes (±10 s), the carrier was put into the Evidence Investigator, and images were captured automatically by the dedicated software.

### 2.7. Image and Data Handling

The chemiluminescent signal from each of the DTRs on the biochip surface was detected with a CCD (charge-coupled device) camera. The dedicated software used image processing to quantify the relative light units (RLU's) and analyte concentration (ppb), and the multiple data generated were processed and archived.

### 2.8. Validation Procedure

The described multi-residue detection method was validated according to the EU instructions for the screening methods (11, 12). For the validation study, the performance characteristics were assessed, including practicability, applicability, specificity, detection capability (CCβ), and stability.

The number of samples required to validate a screening method based on the European guideline (12) depends on the screening target concentration. For setting the screening target concentration at half the regulatory limit or lower (e.g., half of the MRL), at least 20 "screen positive" results are needed to demonstrate that CCβ is less than the Regulatory Limit (MRL) and less than or equal to the half of the MRL. The EU determined safe MRLs of quinolones, including enrofloxacin 100 µg/kg, danofloxacin 30 µg/kg, marbofloxacin 75 µg/kg, ceftiofur 100 µg/kg, thiamphenicol 50 µg/kg, streptomycin and dihydrostreptomycin 200 µg/kg, tylosin and tilmicosin 50 µg/kg, and tetracyclines, oxytetracycline, and chlortetracycline 100 µg/kg in milk (10).

### 2.9. Determining the Cutoff Values and Calculating

When screening, determining a threshold beyond which the sample is categorized as positive is necessary for validating semi-quantitative screening methods (12). The mean and SD of the signal (in RLU) from 20 blank and spiked samples at reported concentrations were calculated for each antibiotic tested. The threshold value T was as below:

T = average RLU signal of blank – 1.64 × SD RLU signal of blank

In addition, the Fm cutoff factor was calculated based on the spiked samples as follows:

Fm = average RLU signal of spiked + 1.64 × SD RLU signal spiked

After calculating the threshold value and the cutoff factor Fm, if the Fm cutoff value was below the positivity threshold T, the target concentration during the validation was selected for CCβ determination. Otherwise, if the cutoff value Fm was not lower than the threshold T, increasing the concentration of antibiotics in the validation step was necessary (Table 1).

**Table 1.** Calibration Range, Maximum Residue Limit (MRL), and Spiking Levels of Six Antibiotics

Compounds	Calibration Range (ppb) with Dilution Factor = 5	MRL (EU) ^a^ (ppb)	Chosen Spike Level (ppb)
**Quinolones (QNL) **	0 - 57.5	-	15
**Ceftiofur (CEFT)**	0 - 35	100	20
**Florfenicol (FFL) **	0 - 25	-	10
**Streptomycin (STR) **	0 - 375	200	100
**Tylosin (TYL) **	0 - 25	50	15
**Tetracycline (TCN) **	0 - 12.5	100	8

^a^ European Union

### 2.10. Practicability, Applicability, and Stability

The purpose of the practicability study was to see if the methodology was suitable for routine investigation. Lack of complication in analyzing, requirements of common lab equipment, and conditions represent the method's practicability. Practicability is not an extra study.

Milk samples representing various degrees of fat content, storage duration, and production place were collected. The method's applicability for screening 6 antibiotic residues was tested by determining the CCβ of different spiked samples in different kinds of milk. The stability of analytes in the solution and the matrix was determined through the literature review.

### 2.11. Presentation of the Method to Real Samples

Thirty-eight UHT milk samples were tested simultaneously for the presence of six antibiotics.

## 3. Results

### 3.1. Detection Capabilities

**Figure 1.**
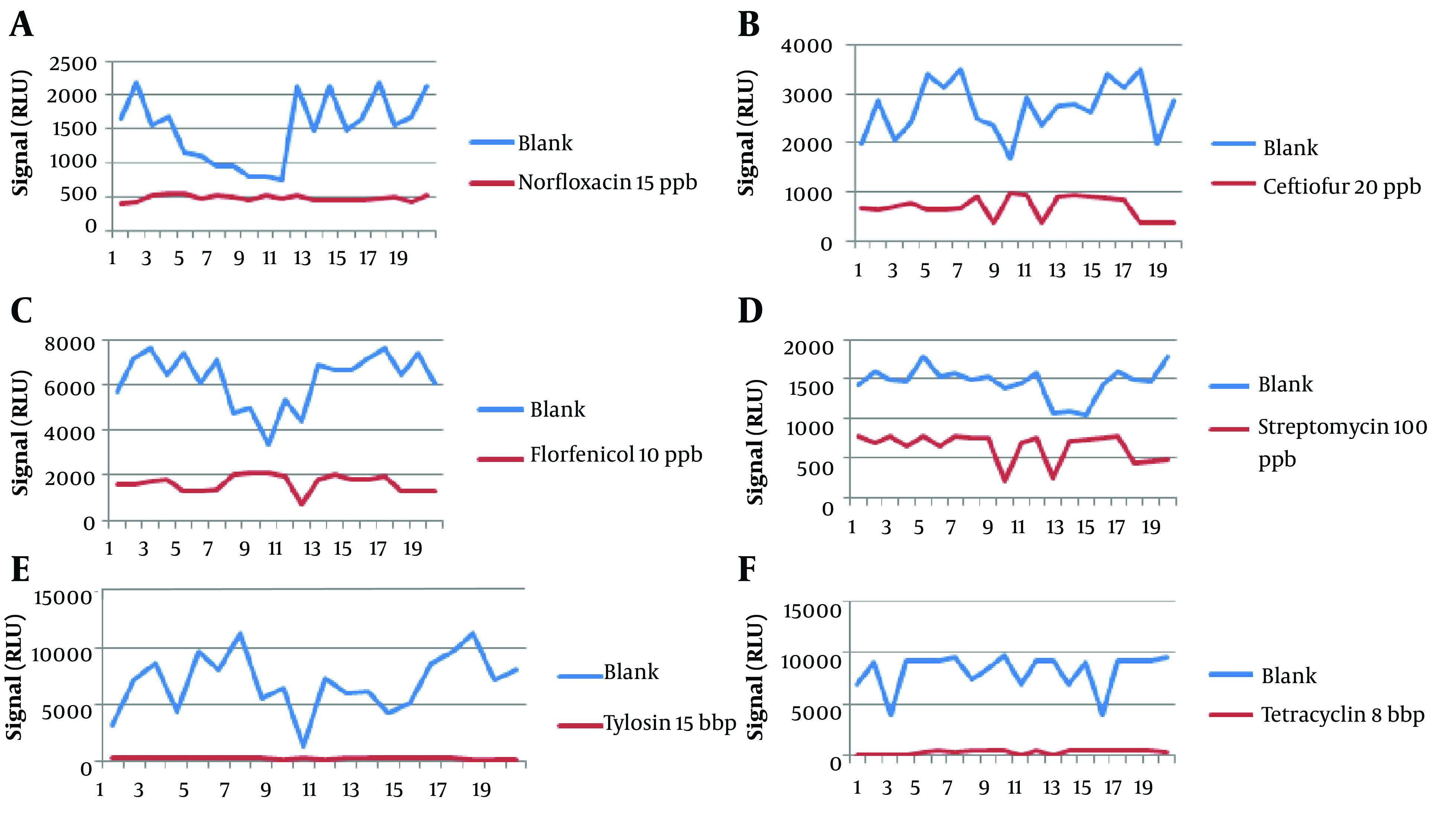
The results of RLU for 20 blank samples and 20 spiked samples for 6 antibiotics

The results from 20 blanks and 20 spiked samples containing 6 antibiotic residues are shown in Figure 1. Table 2 summarizes the results with Fm as the cutoff, and Table 3 shows the CCβ obtained during the validation of 6 antibiotics. The CCβ was defined for 6 antibiotics, norfloxacin, ceftiofur, florfenicol, streptomycin, tylosin, and tetracycline, with no false-negative results. The chosen spike levels (validation concentration) were elected as CCβ because the screening target concentration for authorized analytes is at or below the regulatory limit (MRL) (11).

**Table 2.** Summary of the Fm Cutoff Values

	QNL	CEFT	FFL	STR	TYL	TCN
**Concentration (ppb)**	15	20	10	100	15	8
**T value (RLU)**	686.5396	1807.43	4318.94	1134.549	2673.96	5391.41
**Fm value (RLU)**	557.6017	1049.12	2249.09	933.28	380.34	683.41
**T > Fm**	Yes	Yes	Yes	Yes	Yes	Yes
**Number of FP**	0	0	0	0	0	0
**FP rate %**	0	0	0	0	0	0
**Number of FN**	0	0	0	0	0	0
**FN rate %**	0	0	0	0	0	0

Abbreviations: RLU, relative light unit; QNL, quinolones; CEFT, ceftiofur; FFL, florfenicol; STR, streptomycin; TYL, tylosin; TCN, tetracycline; FN, false negative; FP, false positive.

**Table 3.** Detection Capabilities (CCβ)

	QNL	CEFT	FFL	STR	TYL	TCN
**LOD (ppb) (as per manufacturer)**	1	1.5	0.5	2	2.5	1
**Spike level used for validation (ppb)**	15	20	10	100	15	8
**CCβ (ppb)**	15	20	10	100	15	8

Abbreviations: QNL, quinolones; CEFT, ceftiofur; FFL, florfenicol; STR, streptomycin; TYL, tylosin; TCN, tetracycline; LOD, limit of detection.

### 3.2. Practicability and Applicability of the Kit and Stability of Antibiotic Residues

Practicability is the method's usability and is not a separate study. The purpose of evaluating the practicability of the method is to determine whether it is suitable for routine analysis. The milk samples do not require special preparation, and there is no need to use a large sample volume for the study (only 100 µL). The kit and the software used in this study are both convenient and easy to use.

Storage time of milk and different fat content of samples did not affect the specificity study and CCβ results. Therefore, the Antimicrobial Array II kit is applicable to different kinds of milk samples.

The stability of antibiotic residues in milk has previously been reported in the literature. For example, the stability of quinolones in bovine milk has been investigated, showing that norfloxacin remains stable for 14 days at -20°C (19). Another survey showed that quinolones resist different heat treatments (20). A study investigated the stability of ceftiofur tested every day at 4 ± 2°C and every week at –18 ± 2°C; no obvious changes for 24 – 35 weeks were found (21). The stability of streptomycin milk has been reported for 30 days when stored at -20°C (22). Tylosin has been reported as remaining stable by treatment at 60°C for 30 min (23). Tetracyclines were not degraded after 48 to 55 days at -20°C (24). Another study found that the stability of tetracycline decreased significantly with exposure to 70°C and 100°C for 24 hours, whereas tetracycline was relatively stable over 24 hours at 4°C and 37°C (25).

### 3.3. Specificity and False-Positive Rate

For validation, 20 blanks and 20 spiked milk samples (Table 2) were analyzed over 3 days. While T was considered the threshold, 5% of samples were false-negative for ceftiofur, florfenicol, and tylosin, 10% of samples were false-negative for tetracycline, and 15% of samples were false-negative for streptomycin. No false-positive screening results were obtained. When Fm was considered the threshold, no false-negative or false-positive screening results occurred, as shown in Table 4, these results indicate that when Fm is chosen as the threshold value, the results will be more sensitive, reducing the need for expensive confirmatory analysis.

**Table 4.** The Number of False-Positive and False-Negative Results by the Threshold Value T or Cutoff Factor Fm

Parameter and False-Positive or Negative	Quinolones (QNL)	Ceftiofur (CEFT)	Florfenicol (FFL)	Streptomycin (STR)	Tylosin (TYL)	Tetracycline (TCN)
**T (n = 20)**	686.5396	1807.43	4318.94	1134.549	2673.96	5391.41
**Cutoff = T (n = 20)**						
False-positive	0	0	0	0	0	0
False-negative	0	1	1	3	1	2
**Fm (n = 20)**	557.6017	1049.12	2249.09	933.28	380.34	683.41
**Cut off = Fm (n = 20)**						
False-positive	0	0	0	0	0	0
False-negative	0	0	0	0	0	0

Abbreviations: RLU, relative light unit; QNL, quinolones; CEFT, ceftiofur; FFL, florfenicol; STR, streptomycin; TYL, tylosin; TCN, tetracycline.

### 3.4. Screening of Real Milk Samples

After validation, 38 collected milk samples were screened using the Antimicrobial Array II kit. The results are shown in Table 5. Thirty-one samples were presumptive negative for all the compounds, and seven milk samples were presumptive positive for norfloxacin, streptomycin, or tylosin.

**Table 5.** Incidence of 6 Antibiotics (in RLU) in Ultra-high Temperature (UHT)-treated and Homogenized Milk Samples

	QNL	CEFT	FFL	STR	TYL	TCN
**Number of samples**	38	38	38	38	38	38
**Cutoff (RLUs) **	557.60	1049.12	2249.09	933.28	380.34	683.41
**Number of positive samples**	3	0	0	3	1	0
**% Positive samples**	7.89	0	0	7.89	2.63	0

Abbreviations: QNL, quinolones; CEFT, ceftiofur; FFL, florfenicol; STR, streptomycin; TYL, tylosin; TCN, tetracycline.

## 4. Discussion

Taking antibiotics unnecessarily for treatment and prevention or not observing the required withdrawal periods causes antibiotics to be present in milk (26). The most important health problems caused by consuming milk and dairy products contaminated with antibiotics are allergic reactions and antibiotic resistance (8).

Various tests have been presented so far to evaluate antibiotics in different foods, including milk, with advantages and disadvantages. The choice of analysis method depends on the type of antibiotic, the expected time limits, the sensitivity of the method, and the cost (26). Nowadays, immunological methods are among the most accurate methods used to screen and quantify residual drug compounds in milk and dairy products. These methods are based on the formation of antigen-antibody complexes. Examples of available methods for antibiotic residue screening in milk are shown in Table 6 (27).

**Table 6.** Comparison of Different Commercial Kits or Screening Methods for Antibiotic Residues in Milk

Principle of the Test (Type of Reaction) and Commercial Kit	Number of Tested Antibiotic Residues (6 Antibiotics)	LOD of 6 Antibiotics	Time Per Analysis	Number of Samples at a Time
**Microbial inhibition**				
BRT Inhibitor Test	30 (3)	CEFT:50-100 TCN:200-400 TYL:25-50	2h to 2h and 30 min	Not limited
BRT MRL-Screening Test	30 (3)	CEFT:50-100 TCN:100-200 TYL:25-50	2h to 2h and 30 min	Not limited
Charm Blue-Yellow Test	10 (2)	CEFT:50-100 TYL:75-100	Around 2h and 45 min	Unlimited samples with air incubator
Delvotest P (3 hours)	26 (3)	CEFT:50-70 TCN:200-300 TYL:100-300	2h and 30 min	10 per incubator/water bath, no limit
Delvotest P (control time)	26 (3)	CEFT:<50 TCN:100 TYL:50-100	Starting from 2h and 15 min	10 per incubator/water bath, no limit
Delvotest SP (3 hours)	31 (3)	CEFT:50-70 TCN:200-600 TYL:30-100	3 h	10 per incubator/water bath, no limit
Delvotest SP (control time)	31 (3)	CEFT:<50 TCN:100 TYL:10-20	Starting from 2h and 15 min	10 per incubator/water bath, no limit
Delvotest SP- NT (3 hours)	19 (3)	CEFT:50-100 TCN:800 TYL:50	3 h	10 per incubator/water bath, no limit
Delvotest SP- NT (control time)	19 (3)	CEFT:20-50 TCN:250-500 TYL:30	Starting from 2h and 15 min	10 per incubator/water bath, no limit
ECLIPSE 100	28 (4)	CEFT:100 STR:3000 TCN:150 TYL:80	3.15-3.30 h	No limit, 96 samples per plate
ECLIPSE 50	28 (4)	CEFT:100 STR:2000 TCN:150 TYL:100	2.15-2.30 h	96 samples
Valio T 101 test	32 (3)	CEFT:20-30 STR:1000-1500 TCN:200-300	4 h and 30 min	Depending on incubator
Charm BSDA	9 (2)	CEFT: 150 TYL:200	Around 2h and 45min	Unlimited samples with an air incubator
Copan Milk Test (3 hours)	43 (5)	STR:1000-2000 TCN:250-300 TAF:>100 TYL:50-100	3 h	10 individual tests per dry heat incubator, No limit for incubation in a water bath
Copan Milk Test (control time)	43 (5)	STR:1000 TCN:200 TAF:100 TYL:50	3 h	10 individual tests per dry heat incubator, No limit for incubation in a water bath
KALIDOS MP	26 (3)	STR:800 TYL: 40 TCN:100-150	3 h	Not limited
KALIDOS TB	27 (3)	STR:400-600 TYL:40-50 TCN:100-150	3 h	Not limited
**Lateral flow**				
BETASTAR®	13 (1)	CEFT:75-150	5 min	Up to 6
BETASTAR® COMBO	16)2)	CEFT:75-100 TCN:50	5 min	Up to 6
PENZYM®100	12 (1)	CEFT:40-70	15 min	4
PENZYM®100 S	12 (1)	CEFT:20-40	22 min	4
ROSA MRLBLTET	17 (2)	CEFT:20-50 TCN:7-15	8 min	2 tests per dual incubator,4 tests per quad incubator
Twin sensor BT	17 (2)	CEFT:10-15 TCN:80-100	6 min	8 recommended,48 places on the Heat sensor,incubator
SNAP MRL Beta-Lactam	15 (1)	CEFT:5-13	10 min	One sample per tester, multiple testers operated at the same time by offsetting the start of timing
SNAP Tetracycline	3 (1)	At or below 50	10 min	One sample per tester, multiple testers operated at the same time by offsetting the start of timing
**Radio-labeled Assay**				
Charm II Beta-lactam	12 (1)	CEF:20-40	Approximately 12 min	6 samples per assay
Charm II Amphenicol	4 (1)	TAF:40/50	Approximately 12 min	6 samples per assay
Charm II Macrolide	6 (1)	TYL:50	10-15 min	6 samples per assay
Charm II Tetracycline	3 (1)	TCN:5	12 min	6 samples per assay
Charm II, Aminoglycoside	3 (1)	STR:40	18 min	6 samples per assay
**Solid phase immunoassay**				
Parallux	14 (4)	CEFT:33.7 QNL:20 STR:50 TCN:75	4 min	1-4 samples
**ELISA**				
Streptomycin EIA	1 (1)	STR:4	1.5 h	40 samples in duplicate
Fluoroquinolones EIA	10 (1)	QNL:3	1.5 h	40 samples in duplicate
**Receptor assay**				
Delvo-X-press	22 (1)	CEFT:4-8	3 h	6 per incubator (7 including reference)

Abbreviations: QNL, quinolones; STR, streptomycin; CEFT, ceftiofur; TAF, florfenicol; TCN, tetracycline and TYL, tylosin.

In this survey, the validation of the Antimicrobial Array II kit was based on the European guideline for the validation of screening methods for veterinary medicines (Commission Decision 2002/657/EC) (16, 17). The results indicated that this kit is a valid screening method for the simultaneous determination of the antibiotic residues studied (quinolones, ceftiofur, florfenicol, streptomycin, tylosin, and tetracyclines) in milk samples at the validated concentrations. The CCβ values were under the MRLs authorized by the EC. About 82% of the real milk samples screened were presumptive negative for all the antibiotics. The presumptive positivity needs determination with a confirmatory method. This multiplex biochip-array-based method is semi-quantitative and allows faster and less costly screening analysis, with increasing results output and without requiring highly skilled labor.

Many studies on antibiotic residues in milk samples have been carried out worldwide. In a study conducted in Croatia, out of 1,259 milk samples, it was determined using an immunoassay method that only one sample was contaminated with tetracycline (28). In another study in Macedonia, 13.1% and 6.8% of samples were contaminated with tetracycline and quinolones, respectively (29). In a recent survey conducted in Delta state, Nigeria, out of 126 fresh milk and 79 fermented milk samples tested for tylosin, 24% and 11% were positive, respectively (30). Twenty-five milk samples from Central California were tested for ceftiofur, and 7 were positive (31). Other investigations showed a higher number of samples studied for antibiotic residues. For example, 17 out of 36 milk samples studied were positive for tetracycline in a survey conducted using both lateral flow immunochromatography assay based on up-converting nanoparticles and High-performance Liquid Chromatography (HPLC) methods (32). In Central New York State, 34 waste milk samples were analyzed by enzyme-linked receptor-binding assay, and 75% contained beta-lactams, 14.3% contained tetracycline, and 7.1% contained sulfamethazine residues (33). In another study, where 22 milk samples were tested by the ELISA method, 86.4% were positive for tetracycline (34).

In Iran, 187 milk samples were tested by HPLC under isocratic conditions using UV detection, and it was reported that only 2 samples were positive for tetracycline (35). Another study tested 15 bovine milk samples; only 1 was contaminated with florfenicol (36). In a survey, 90 pasteurized and 14 raw milk samples were analyzed for tetracycline residues; 5.6% and 7.1% were contaminated, respectively (37). In a study conducted in Tehran, out of 240 milk samples, 72 and 42 were positive for beta-lactam and tetracycline, respectively, and 18 samples were contaminated with both (38). Another survey showed that of 251 milk samples analyzed for β-lactam and tetracycline antibiotic residues by the Copan test, 62 (24.8%) were positive (39). In another study, β-lactam and tetracycline were determined in 848 milk samples collected from West Azerbaijan province by Copan test kit, and 30.14% contained antibiotic residues (40).

The variety of screening methods available and the increasing number of studies in this field show the importance of this topic worldwide. Although in this report, the results showed few presumptive positive samples, extensive testing is needed to identify more antibiotic residues in various ranges of dairy products in different provinces of Iran. The method validated in this report increases the screening capacity without compromising the analytical performance and provides a sensitive, rapid, and useful screening tool for simultaneously detecting 6 antibiotic residues in different milk types.

### 4.1. Conclusions

In modern agriculture, the irrational or illegal use of veterinary medicines leads to residues in animal foods that can seriously endanger human health and cause financial losses in the dairy industry. A key challenge for analytical methods is the efficient detection of low concentrations of drug residues in animal products in a short time. To our knowledge, this is the first study to validate the Antimicrobial Array II kit in milk according to Commission Decision 2002/657/EC and the European Directive on validating veterinary medicinal product screening methods. This method can simultaneously screen 6 antibiotics in milk samples with optimal analytical performance quickly, with easy preparation and lower cost compared to chromatographic methods.

Although in this survey, ceftiofur, florfenicol, and tetracycline were not found above the EU MRLs in any of the samples, and less than 8% of the samples were contaminated with each of the other antibiotics mentioned, further studies on these antibiotics in different types of milk need to be carried out. More samples are needed and recommended, as well. The drugs used in livestock farming must be strictly controlled. Ranchers should be made aware of the negative public health impacts of uninformed use of antibiotics, and training programs should be prioritized.
